# Lemur Biorhythms and Life History Evolution

**DOI:** 10.1371/journal.pone.0134210

**Published:** 2015-08-12

**Authors:** Russell T. Hogg, Laurie R. Godfrey, Gary T. Schwartz, Wendy Dirks, Timothy G. Bromage

**Affiliations:** 1 Department of Rehabilitation Sciences, Florida Gulf Coast University, Fort Myers, Florida, United States of America; 2 Department of Anthropology, University of Massachusetts at Amherst, Amherst, Massachusetts, United States of America; 3 Institute of Human Origins and School of Human Evolution and Social Change, Arizona State University, Tempe, Arizona, United States of America; 4 Department of Anthropology, Durham University, Durham, United Kingdom; 5 Department of Biomaterials and Biomimetics, and Department of Basic Science and Craniofacial Biology, New York University College of Dentistry, New York, New York, United States of America; Monash University, AUSTRALIA

## Abstract

Skeletal histology supports the hypothesis that primate life histories are regulated by a neuroendocrine rhythm, the Havers-Halberg Oscillation (HHO). Interestingly, subfossil lemurs are outliers in HHO scaling relationships that have been discovered for haplorhine primates and other mammals. We present new data to determine whether these species represent the general lemur or strepsirrhine condition and to inform models about neuroendocrine-mediated life history evolution. We gathered the largest sample to date of HHO data from histological sections of primate teeth (including the subfossil lemurs) to assess the relationship of these chronobiological measures with life history-related variables including body mass, brain size, age at first female reproduction, and activity level. For anthropoids, these variables show strong correlations with HHO conforming to predictions, though body mass and endocranial volume are strongly correlated with HHO periodicity in this group. However, lemurs (possibly excepting *Daubentonia*) do not follow this pattern and show markedly less variability in HHO periodicity and lower correlation coefficients and slopes. Moreover, body mass is uncorrelated, and brain size and activity levels are more strongly correlated with HHO periodicity in these animals. We argue that lemurs evolved this pattern due to selection for risk-averse life histories driven by the unpredictability of the environment in Madagascar. These results reinforce the idea that HHO influences life history evolution differently in response to specific ecological selection regimes.

## Introduction

Growth, metabolism, and reproductive physiology all have a role to play in the allocation of resources over individual lifespans, and have all been implicated in multiple explanatory models of life history evolution (e.g. [1–6]). While many of the effects of ecology on life history have been understood for some time, the manner in which the evolution of physiological systems is coordinated to achieve particular life history outcomes remains unclear. Recently, Bromage et al. [2,3,7] used histological evidence from primate dental and osseous tissues to address this question. Using vitally labeled bone to assess the chronology between growth increments in bones (lamellae), they showed that lamellae are laid down with a specific periodicity, which ranged from 1 day in rats to 8 days in humans. Moreover, they demonstrated that the lamellar periodicity matches that of long-period growth increments in tooth enamel, known as striae of Retzius. This periodicity occurs as a multiple of whole days and is variable among and within some taxa, but not within individuals. Mammalian periodicities range between 1 and 14 days [8,9], and manifest as daily increments between adjacent striae of Retzius, known as cross striations. While dental incremental growth lines have long been recognized as periodic structures [10], the recognition that lamellae are periodic is new.

Although bones and teeth are both mineralized tissues, they have different cellular and developmental origins; dental tissues, in fact, have origins from both ectoderm-derived epithelium (enamel) and mesenchyme (dentine), and both express long-period growth lines (called Andresen lines in dentine, while von Ebner lines between them are daily) [7]. The synchronization of enamel, dentine, and bone suggests a central mechanism operating systemically to regulate tooth and bone development as well as the growth of other organ and tissue systems. Bromage et al. term this common biorhythm the Havers-Halberg Oscillation (HHO), and argue that it is controlled centrally by the hypothalamus via oscillations in sympathetic nervous output [2,3]. These oscillations are hypothesized to control cell proliferation and thus growth rates, metabolic rates, and body mass. If this is the case we would expect the HHO to have an expression in soft tissues as well as mineralized tissues, and several studies have provided evidence that this is the case. For example, in 2007 Brown et al. [11] demonstrated that mammal fibroblasts from different species lose the allometric scaling of their metabolism when removed from the physiological context of their host organism, and begin to behave similarly to one another; in other words, metabolic rates of *in situ* fibroblasts scale with body mass across species, whereas metabolic rates of explanted fibroblasts from the same species do not scale. This suggests that a central physiological mechanism is responsible for coordinating metabolic function among the cells that make up an organism. It has also been demonstrated that all primate tissue and organ masses necessarily covary with body mass, and thus also scale with HHO periodicity in a manner similar to one another [12]; this indicates the HHO's general applicability to most of the body's cellular milieu. Bromage et al. [3] have also assessed HHO with regard to temporal life history variables and have shown a link between HHO periodicity and estrous cycling in anthropoids, and have noted several potential physiological pathways through which the HHO can affect metabolism, growth, and reproduction.

Accordingly, the HHO would act as a metronome around which life history physiology is organized. Following this model, high-frequency HHO oscillations (e.g. 2–3 days) will foster more rapid cell proliferation and faster overall life histories linked to smaller body size, while low-frequency HHO oscillations (e.g., 8–9 days) will have the opposite effect. A more detailed discussion of the physiology putatively underlying the HHO mechanism and the evidence to support it may be found in studies by Bromage et al. [2,3].

Following the HHO hypothesis, interspecific HHO periodicity should correlate positively with body mass, brain mass, and basal metabolic rate. Likewise, the HHO should also relate to tissue specific metabolic rates, inasmuch as they aggregate in rational proportion to whole-body metabolic rate. A proxy for bone tissue specific metabolic rate is osteocyte lacuna density (the number of osteocytes per unit volume of bone), which should correlate negatively with body mass, as smaller species should have higher-frequency HHO and therefore more cell proliferation rhythms per unit time. Prior studies have corroborated the HHO periodicity predictions for anthropoid primates and osteocyte densities predicted for mammals [2,3,7].

The HHO mechanism holds great promise for the development of powerful explanatory models of life history evolution. However, the predictive power of HHO-based models is currently limited by a lack of information on how HHO periodicity varies among taxa, and a lack of information on how this variation may be influenced by differing ecological selection regimes and phylogenetic histories. The insular lemurs of Madagascar (Lemuroidea and Daubentonioidea, Lemuriformes, Strepsirrhini, Primates) provide an excellent opportunity to fill some of these gaps, since they exhibit major life history differences from their relatives, the monkeys and apes (Anthropoidea, Haplorhini, Primates) [1]. With the exception of the aye-ayes (*Daubentonia*, Daubentonioidea), they also have reduced activity levels, smaller home ranges, and smaller brains compared to like-sized anthropoids [6]. However, very few strepsirrhine primates were included in previous HHO studies, and data from these few genera conflict across different analyses [2]. Importantly, analyses of the relationship of HHO periodicity to body mass have included only the loris *Nycticebus* and the giant extinct lemurs *Megaladapis* and *Palaeopropithecus*, the latter two of which have a very short HHO periodicity relative to their body size as compared to all anthropoids. This anomaly suggests that the giant lemurs may differ from other primates in HHO biology. As a second possibility, it may be that the lemurs in general differ from other primates, or third that the strepsirrhines as a whole follow this pattern. On the basis of prior osteocyte density data [2], we predict this third option is much less likely to be the case, but it cannot be ruled out *a priori*. While an unusual lemur HHO biology is not entirely surprising given the unusual nature of life histories in this group, it behooves us to examine the strepsirrhines further to clarify their patterns of life history evolution. Doing so will also inform our understanding of life history evolution among primates and mammals in general, and possibly develop a more nuanced understanding of the conditions under which the HHO may evolve. Metabolic flux is key to an organism's life history adaptation to its ecology [13]; thus while no study on how the HHO may evolve in response to ecological differences has been made, we hypothesize that answers will relate to metabolism.

This study examines the relationships among HHO and life history-related variables for the largest sample of primate taxa analyzed to date, including a greatly expanded strepsirrhine dataset. We predict that HHO periodicity values seen previously in giant lemurs will extend to the lemurs in general, which will differ from anthropoids in the expression of their HHO biology due to differences in their physiology, life history, and ecology. Since strepsirrhines as a whole may share aspects of their life history space that are absent in anthropoids, as stated above it is alternatively possible that differences extend to the non-lemur strepsirrhine superfamilies (Lorisoidea and Adapoidea). We also expect that when HHO periodicity is regressed against metabolic rate, differences seen among taxa will be less pronounced than they are for body mass and brain mass. This is due to the fact that metabolism is a more direct putative target for HHO regulation than is body or brain size, both of which may be subject to more diverse physiological influences.

## Materials and Methods

This study utilized only skeletonized specimens of previously deceased animals, acquired from museum and university collections. No living animals were included in the study, and no living animals were affected in any way for the purpose of this study. Sources and specimens used are listed as follows: University of Massachusetts uncatalogued *Avahi laniger*, Jonah Ratsimbazafy Collection, Manombo Reserve; Duke Lemur Center: DLC 11832, "Poe", 6514m, 4564m, 6845m, 5628f; Great Divide Basin Project: WMU VP 359, 654826, 6541103, 4587, 371, 363, g501589; University of Massachusetts Anthropological Primate & Natural History Collection: UM-APC 27, 9, 253, 170, 163; Collection of Anthony DiFiore, PhD, University of Texas: 2 uncatalogued *Lagothrix poeppiggii*; Newcastle University School of Dental Sciences: HT 13–90, 16–90, 25–08, 01–10, 06–02, 07–02, 19–05, 12–90, 17–00; Arizona State University: S26; Montpelier II University: ACQ 6415; Universität Zürich-Irchel: Co.p 3. More detail on specimens and their sources is available in the supporting information (S1 Table).

### (a) Dental sample and protocols

HHO cycle duration was quantified as the period, in days, between successive striae of Retzius (Retzius period, RP) in teeth of 41 anthropoid and 21 strepsirrhine species. We are introducing the term RP as a synonym and replacement for “periodicity” and “repeat interval” (RI) used by authors in prior studies (e.g., [2,3,14–16]) in an attempt to establish a unified terminology; we argue that the prior terms lack specificity and do not sufficiently address the oscillatory nature of HHO physiology, respectively. RP data were gathered from our own histological preparations or from compilations in the literature [2,3,7]. All teeth that we examined directly were cleaned, embedded in acrylic resins, sectioned, and polished using standardized dental histology protocols [7,14,15], and were imaged using transmitted polarized light microscopy. All dental data taken from our own specimens (see S1 Table) and from the literature are listed in S2 Table.

**Fig 1:**
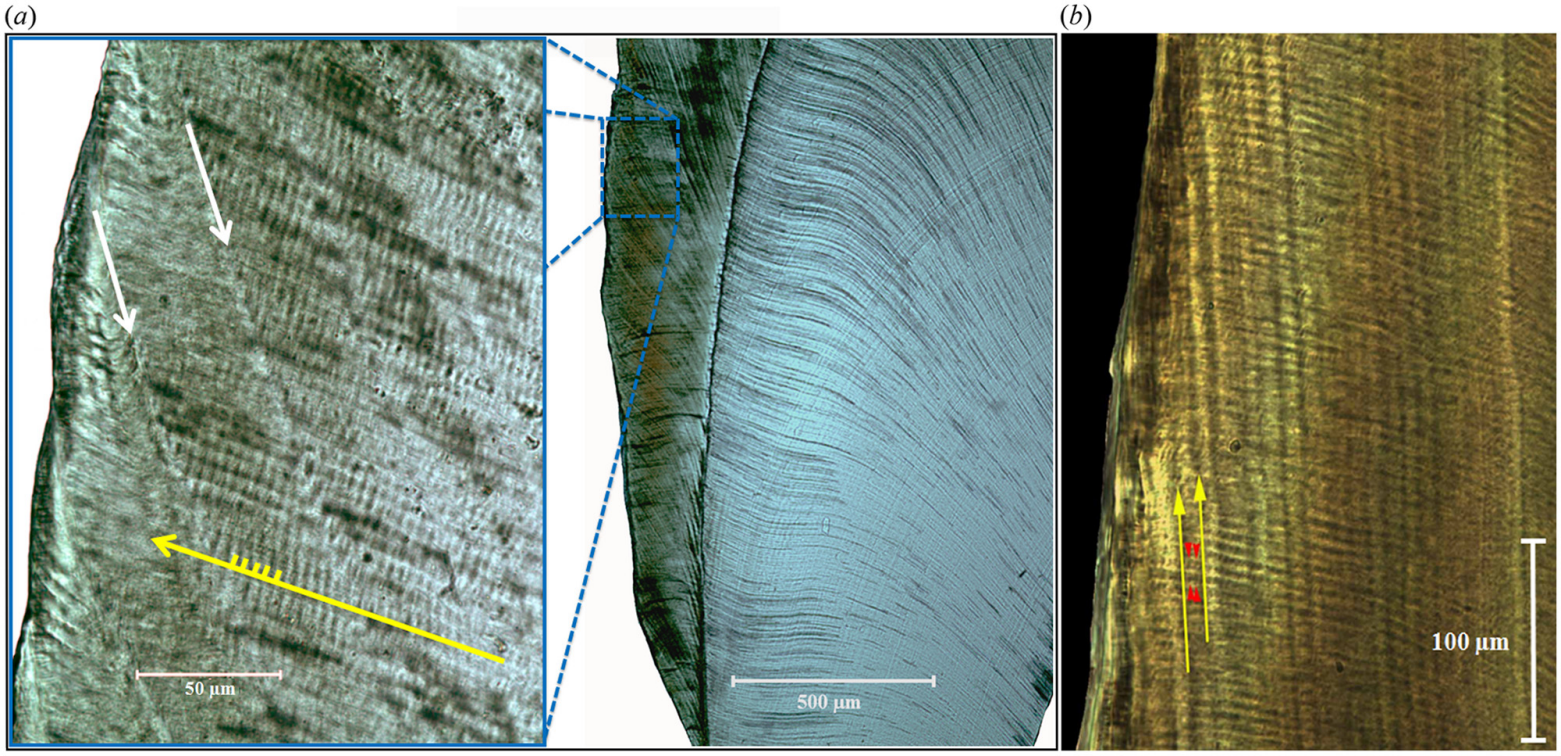
Appearance of tooth enamel in histological sections. (*a*) An orangutan (*Pongo pygmaeus pygmaeus*) molar. Dentine is toward the right, enamel surface to the left. Cusp tips are toward the top. In the inset (adapted from [16]), individual enamel prisms (yellow arrow) run from the dentine to the enamel surface, with daily cross striations (yellow hash-marks) running across prism long axes. Striae of Retzius (white arrows) run obliquely from outer enamel to the enamel-dentine junction. RP (11 days here) can be determined by counting cross striations between successive striae of Retzius. (*b*) A diademed sifaka (*Propithecus diadema*) molar, whose RP = 3 days (yellow arrows = striae of Retzius, with 3 cross-striations visible between them; red arrows demarcate transition between striations).

RP values were determined from histological images in one of two ways. First, cross-striations (24-hour growth increments) between successive, periodic striae of Retzius were counted directly at several locations within a tooth (Fig 1). Second, measurements of enamel prism lengths between successive striae of Retzius were made and then divided by the average daily secretion rate of enamel (calculated across multiple cross-striations) in that area of the crown.

### (b) Statistical analyses

Natural logarithm (ln) of RP mean (mode for species where n > 3) for each species were regressed against ln of data from the literature for body mass, endocranial volume (ECV, as a proxy for brain mass), and age at first female reproduction (AFR, as one chronometric assessment of life history) [2,14,15,17–37]. Metabolism is also a critical variable to consider; however, it cannot be directly measured in an important subset of lemurs—the large-bodied extinct ones. Spoor et al. and Walker et al. [29,30,38] have shown that semicircular canal radius (SCR) is a reliable proxy for activity levels in extinct and extant organisms [6]. Moreover, the relationships between SCR and BMR for extant anthropoids and strepsirrhines are very strong and significant (r = 0.920, p < 0.001, n = 12 and r = 0.968, p = 0.001, n = 10, respectively). Therefore, we use ln SCR in our analyses as a metabolic proxy variable. For a few analyses we also calculated index of cranial capacity (ICC), following the formula from Martin, modified to incorporate the exponent given in Isler et al. (ICC = ECV / body mass^0.646^) [25,28] and including all of the body mass and ECV values of our dataset as cited above and listed in S2 Table. ICC is an encephalization statistic that calculates relative brain size, accounting for the degree to which brain mass covaries with body mass. ICC is not logarithmically transformed in our analyses as are other variables, since it is an index.

The natural logarithms (ln) of body mass, ECV, SCR, and AFR were analyzed against ln RP using bivariate phylogenetic generalized least-squares (PGLS) regression [39] using the caper package for R (URL: http://www.R-project.org/), in order to control for phylogenetic effects. Consensus trees for these analyses were generated using 10K Trees (URL: http://10ktrees.fas.harvard.edu/) except for lemurs, whose phylogeny was taken from Catlett et al. [18] and Kistler et al. [40] to include the subfossil taxa. To incorporate subfossil lemurs, branch lengths were calibrated as a chronogram. For all PGLS analyses, we also used Pagel’s Lambda (λ) as a statistic denoting the extent of phylogenetic signal, or the degree to which trait covariation reflects their shared evolutionary history (as approximated by Brownian motion); at λ = 0, phylogenetic relatedness of species has no correlation with trait covariation, and thus the trait of interest may vary randomly across a phylogeny while at λ = 1, closely related species tend to exhibit more similarity in trait expression [41].

Our raw data table (S2 Table) and cladogram (S1 Fig) are available in the supporting information. Non-phylogenetically-corrected bivariate regression results are also available in S3 Table.

## Results

**Fig 2:**
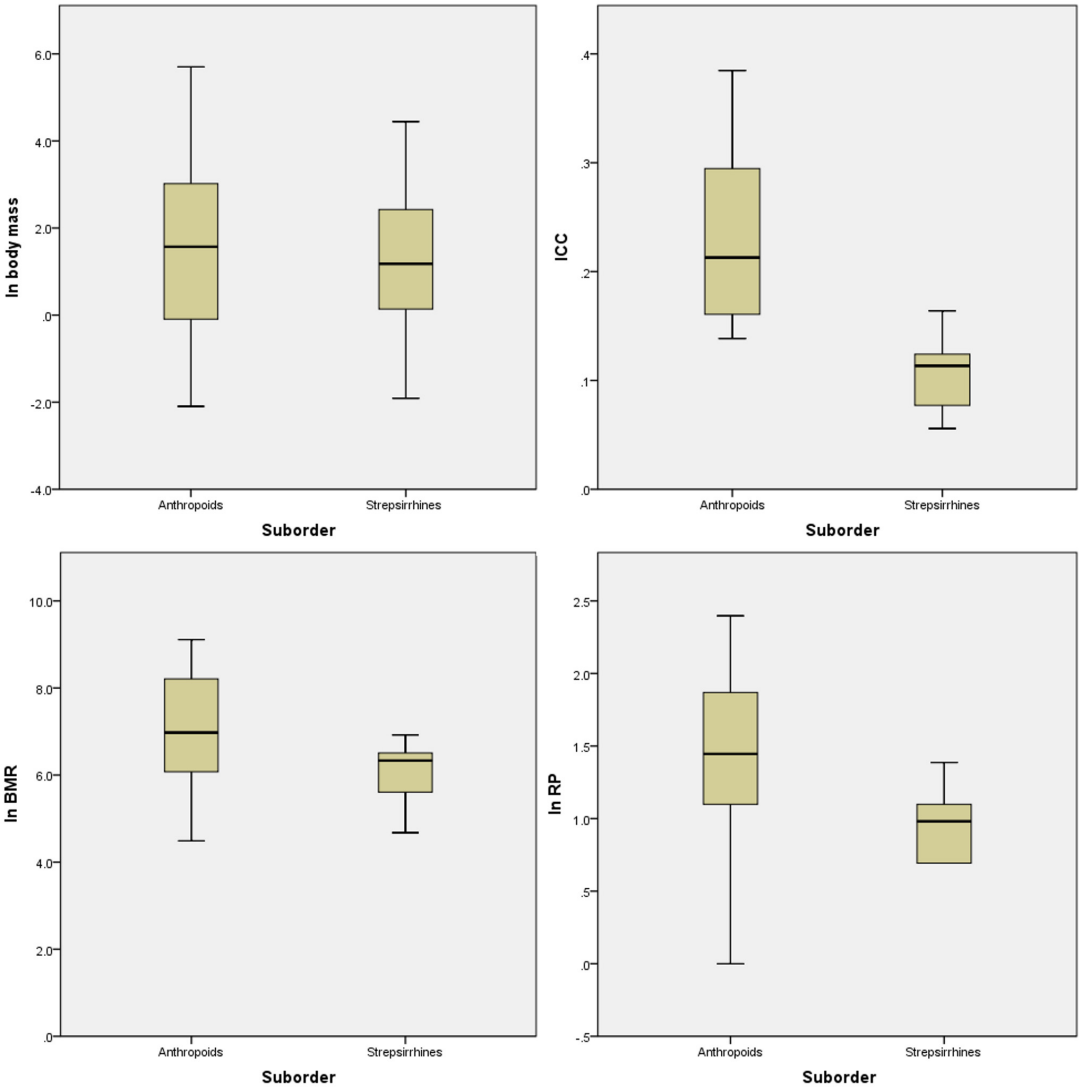
Variation of life-history related variables in strepsirrhines. Standard boxplot demonstration of variation in ln body mass, basal metabolic rate (BMR), index of cranial capacity (ICC, see methods), and Retzius periodicity (RP) between the extant anthropoids and strepsirrhines sampled in this study. ICC is a measure of relative brain size, or encephalization, as opposed to ECV which measures absolute brain size; we have focused on encephalization in this chart to compare anthropoid and strepsirrhine brain size variation because absolute brain size covaries with body size. All plots are calculated using data in S2 Table (supporting information). Not all species for this study are included in all plots, as data for some species are missing for particular variables (especially BMR). S2 Table details which species had data available for them for the four variables.

Overall, strepsirrhines have a high degree of variation in body mass, on a level comparable with the anthropoids (Fig 2). However, they exhibit a greatly restricted range of variation in BMR as well as encephalization compared to the anthropoids, and they exhibit an especially restricted range of variation in RP.

**Fig 3:**
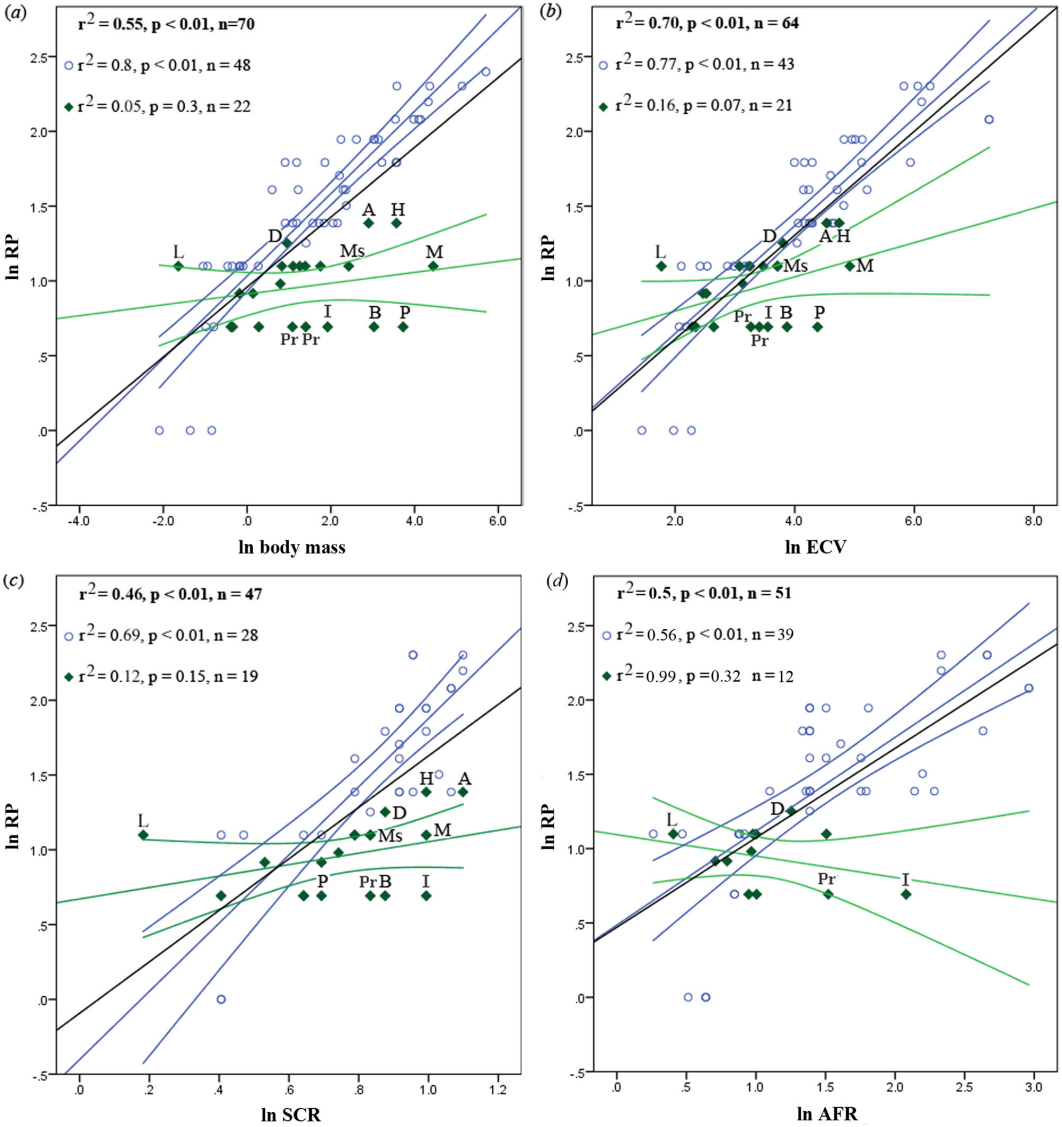
Bivariate regressions of ln RP, raw values (without phylogenetic correction), including 95% confidence intervals. (*a*) Versus ln body mass, (*b*) ln ECV, (*c*) ln SCR, and (*d*) ln AFR. Black lines & bold print indicate regression relationships for the whole primate sample; blue circles & lines represent anthropoids, green diamonds and lines represent strepsirrhines. A = *Archaeolemur majori*, B = *Babakotia radofilai*, D = *Daubentonia madagascariensis*, H = *Hadropithecus stenognathus*, I = *Indri indri*, L = *Loris tardigradus*, M = *Megaladapis edwardsi*, Ms = *Mesopropithecus globiceps*, P = *Palaeopropithecus ingens*, Pr = *Propithecus verreauxi* & *diadema*.

Phylogenetically corrected bivariate analysis results are available in Tables 1–3. For visualization purposes, we have also included raw (uncorrected) bivariate regression plots in Fig 3, and have included associated statistics in S3 Table. PGLS and raw results are very similar to one another. In general, our analyses show major differences in HHO scaling between anthropoids and strepsirrhines.

**Table 1:** PGLS bivariate regression statistics, anthropoid sample. λ represents the degree to which the covariation between the two variables is influenced by phylogeny. Significant relationships highlighted in bold. Multivariate analysis includes estimates of each independent variable’s importance for predicting the dependent variable using Akaike’s Information Criterion (with correction for small sample size, AICc) weight. Tables 2 and 3 display the same information for different sample sets.

Anthropoids (Bivariate Analyses, PGLS):							
	Model	λ	r^2^	p	n	slope	std. error
ln RP vs.	***ln Body Mass***	***0*.*170***	***0*.*720***	***<0*.*01***	***35***	***0*.*323***	***0*.*036***
	***ln ECV***	***0***	***0*.*766***	***<0*.*01***	***35***	***0*.*371***	***0*.*036***
	***ln SCR***	***0***	***0*.*677***	***<0*.*01***	***25***	***2*.*163***	***0*.*318***
	***ln AFR***	***0***	***0*.*528***	***<0*.*01***	***31***	***0*.*622***	***0*.*109***

**Table 2:** PGLS bivariate regression statistics, strepsirrhine sample.

Strepsirrhine (Bivariate Analyses, PGLS):							
	Model	λ	r^2^	p	n	slope	std. error
ln RP vs.	ln Body Mass	0	0.062	0.310	20	0.037	0.035
	ln ECV	0	0.189	0.060	19	0.119	0.060
	ln SCR	0	0.120	0.150	19	0.383	0.252
	ln AFR	0	0.099	0.320	12	-0.144	0.137

**Table 3:** PGLS bivariate regression statistics, lemur sample.

Lemurs (Bivariate Analyses, PGLS):							
	Model	λ	r^2^	p	n	slope	std. error
ln RP vs.	ln Body Mass	0.784	0.130	0.187	15	0.074	0.053
	***ln ECV***	***0*.*436***	***0*.*302***	***0*.*030***	***15***	***0*.*197***	***0*.*083***
	***ln SCR***	***0*.*416***	***0*.*343***	***0*.*020***	***15***	***1*.*116***	***0*.*429***
	ln AFR	0	0.072	0.490	9	-0.157	0.214

Among anthropoids, results are consistent with prior studies in that ln body mass and ln ECV both show strong and significant relationships with RP (Table 1). Although ln ECV is the strongest predictor, it is followed closely by ln body mass. Both variables share a similar slope with regard to ln RP, therefore it seems they are both part of a covarying suite of characters forming a strong relationship with ln RP and life history. Furthermore, ln SCR and ln AFR also have significant correlations with ln RP among the anthropoids.

Strepsirrhines, however, do not show strong correlations between the predictor variables we examined and ln RP (Table 2). When the complete strepsirrhine sample is considered, there are no significant predictors for ln RP, although ln ECV approaches significance. Within the lemur sample, ln ECV and ln SCR both are significant predictors, however their r^2^ values are not high (0.302 and 0.343, respectively; λ values for both variables are not significantly different from 1; Table 3).

These weak correlations between our predictor variables and ln RP are best explained as a consequence of such low variation in ln RP among the strepsirrhines in general and the lemurs in particular. Or, in other words, given the same RP value for a strepsirrhine as for an anthropoid, we see a much greater range of variation in the predictor variable values among the strepsirrhines. This is especially the case for the lemurs, since the non-lemur strepsirrhine species in our analyses all have small body sizes and a low variation in their range of body sizes, and indeed fall near the intersection of the anthropoid and strepsirrhine regression lines. So while it is difficult to discern a pattern in the non-lemur strepsirrhines given the data we have, the plots make it very clear that the original, unusual RP pattern noted for *Megaladapis* and *Palaeopropithecus* is not restricted to the large subfossil taxa, but instead extends to the lemuroids in general. For example, indriids do generally fall further from the strepsirrhine and primate regression lines than lemurids (Fig 3), but the female-gorilla-sized *Megaladapis* (extinct sister taxon to lemurids) also falls well below the line.

**Fig 4:**
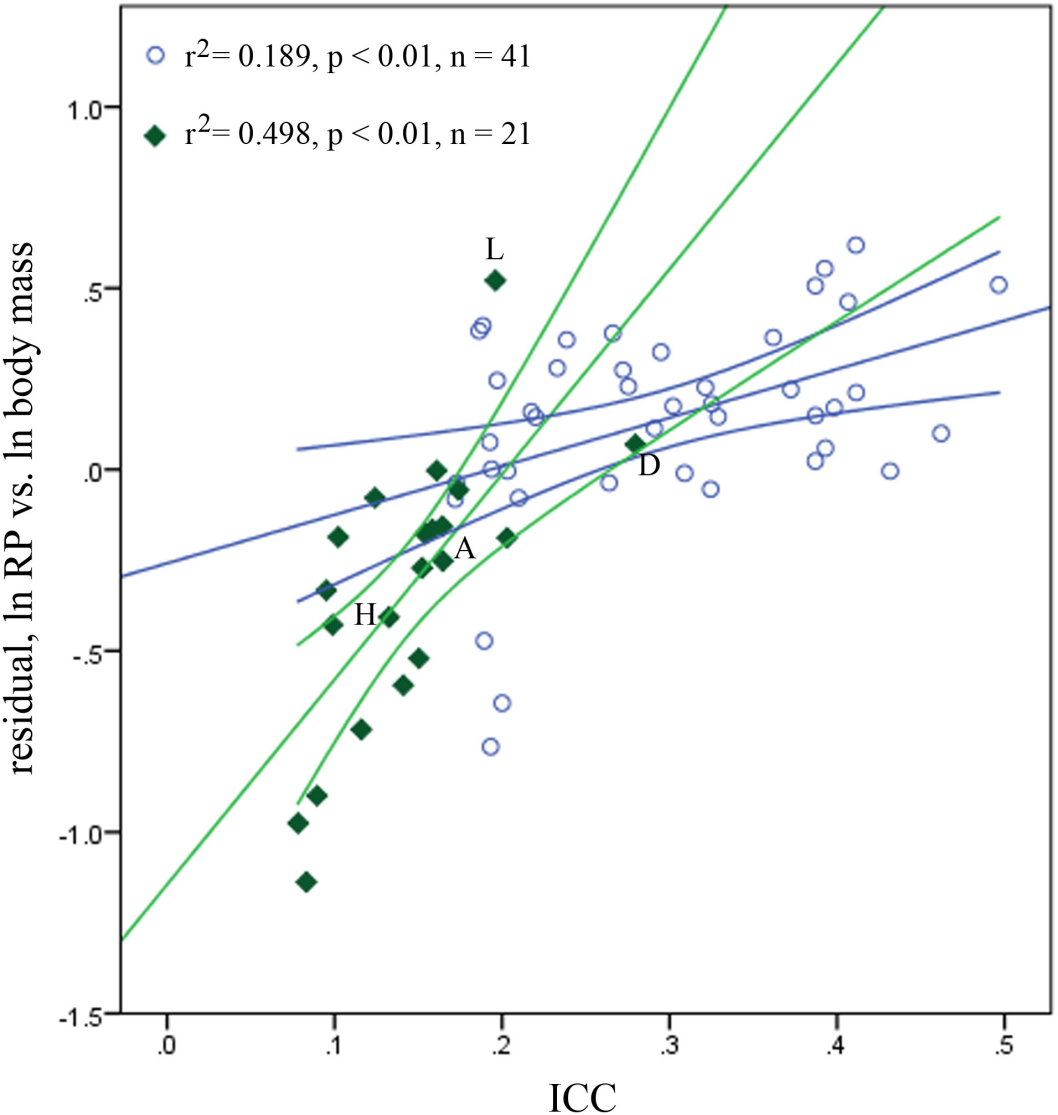
Bivariate regression of ln RP residuals (without phylogenetic correction, regressed against ln body mass) vs. ICC, including 95% confidence intervals. Blue circles & lines represent anthropoids, green diamonds & lines represent strepsirrhines. This chart corrects for effects of body size on both RP and brain size, to visualize relationships between ln RP and encephalization. A = *Archaeolemur majori*, D = *Daubentonia madagascariensis*. H = *Hadropithecus stenognathus*, L = *Loris tardigradus*.

However, there are several notable outliers in our analyses (Fig 3). Among the lemurs, *Archaeolemur*, *Hadropithecus*, and *Daubentonia* consistently have the highest positive residuals and therefore resemble anthropoid patterns more than other lemurs do—particularly *Daubentonia madagascariensis*. This species is a particularly interesting case, as it is exceptional among lemurs due to its high encephalization and relative BMR, and unique life history profile. Since *Daubentonia* is unusually encephalized, we wanted to visualize the position of *Daubentonia* compared to the anthropoids and other strepsirrhines in a manner that would control for the effect of body size on brain size and ln RP. Therefore, we performed an analysis of ln RP residuals (from a regression against ln body mass) against index of cranial capacity (ICC, see Fig 2) as a means of visualizing the relationship between ln RP and brain size independent of their connections to body size (Fig 4). While both strepsirrhines and anthropoids exhibit a statistically significant relationship between ICC and ln RP residuals in this analysis, the strepsirrhines exhibit a very different pattern that includes a much stronger correlation (again, see Fig 4). This provides additional evidence that body mass may hold a different biological connection to RP in anthropoids versus strepsirrhines. More pertinent here, however, is the fact that *Daubentonia* falls very near the anthropoid regression line, well away from the intersection of the strepsirrhine and anthropoid lines. Taken in addition to results from raw bivariate regressions, this suggests that of all the lemurs, *Daubentonia* stands out as the most anthropoid-like in its RP evolution.

It is also not surprising that *Archaeolemur* and *Hadropithecus* appear as outliers in our regressions (although they are not outliers in Fig 4), as they have been reconstructed as having fairly unusual life histories among subfossil lemurs (see discussion below). In contrast, it is noteworthy that the indriid and palaeopropithecid lemurs *Indri*, *Propithecus*, *Palaeopropithecus*, and *Babakotia* fall out consistently far below the strepsirrhine regression lines, with the two extinct palaeopropithecids (*Palaeopropithecus* and *Babakotia*) possessing an especially “hyper-strepsirrhine” pattern when assessed against ln body mass and ln ECV. *Loris tardigradus* is also interesting in that it generally falls well above all other similar-sized primates in all graphs, with the exception of ln AFR (Fig 3). Known for its low activity levels, this genus has a low SCR for its body mass when plotted on a linear regression in natural log-transformed space. For primates as a whole, when SCR is regressed against body mass without logarithmic transformation a curvilinear relationship appears, with smaller primates having more variation in relative SCR size; even among these small primates, *L*. *tardigradus* falls at the bottom of the curve.

## Discussion

Strepsirrhines consistently show scaling relationships for HHO that diverge from those of the anthropoids. The most obvious pattern is that anthropoids possess a broad range of mean RP values (and therefore HHOs) from 1 to 11 days, whereas strepsirrhines range only between 2 to 4 days. A comparison is useful: one of the largest extinct lemur species, *Megaladapis edwardsi*, has an RP of 3 days whereas comparably-sized anthropoids such as female *Gorilla* have modal RPs of 9. The limited range of strepsirrhine RP values levels the slopes of their regressions, reduces the strength of correlations, and suggests that lemurs in particular (or strepsirrhines in general) have an HHO biology that is more constrained than that of anthropoids.

**Fig 5:**
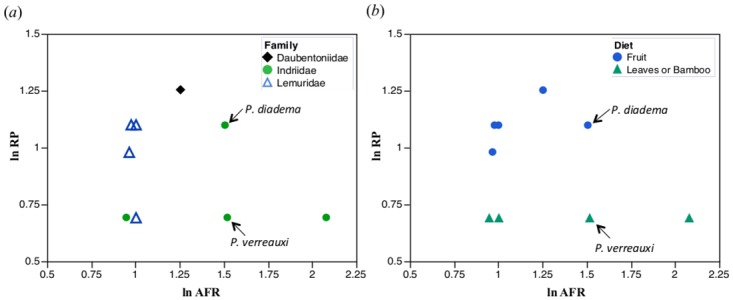
Bivariate plots of ln RP vs. ln AFR in extant lemurs. (*a*) Separated at the rank of family, and (*b*) separated by diet.

Furthermore, the relationships between HHO periodicity and our predictor variables are markedly different between anthropoids and strepsirrhines. Anthropoids show a strong linear relationship between ln RP and all predictor variables, particularly ln ECV and ln body mass, as we would predict from prior studies. In sharp contrast, variation in ln RP is poorly explained in lemurs by any single variable, mostly because the variance in ln RP among lemurs is very limited. As a clade, lemurs have relatively small brains. They are also hypometabolic and RP is low, while other variables such as body mass and age at first reproduction vary tremendously. The Indriidae, for example, can delay first reproduction long after weaning, and species with remarkably late ages at first reproduction are among those taxa with the lowest values for RP. In other words, indriids can delay age at first reproduction with no effect on RP (which remains low). This is not the case for lemurids, where one sees an opposite pattern: age at first female reproduction is early, regardless of RP (Fig 5a).

For both Indriidae and Lemuridae, there appears to be a dietary effect, wherein species that consume more fruit have higher RP values, regardless of age at first reproduction (Fig 5b). Thus *Hapalemur griseus* (a small lemurid that specializes on bamboo) has an early age at first female reproduction and low RP, while the largest-bodied extant lemurid, *Varecia*, which is highly frugivorous, has relatively high RP but also begins reproducing early. *Propithecus diadema*, probably the most frugivorous indriid, has higher RP values than the more folivorous *Indri indri* and *P*. *verreauxi* (which begin reproducing slightly to considerably later), and it has higher RP values than *Avahi laniger* (which begins reproducing considerably earlier). The daubentoniid *Daubentonia madagascariensis* has the highest RP value among extant lemurs (as well as relatively large brains and high activity levels), and a rich diet of structurally defended fruit and animal matter. Age at first reproduction is early in comparison to *Indri*, but delayed in comparison to lemurids.

The dietary constraint that we have identified here may hold for the extinct lemurs, as *Palaeopropithecus* especially but also *Babakotia*, *Mesopropithecus*, and *Megaladapis* all have RP values of 2 or 3 (regardless of body size), and diets that were highly folivorous. The extinct lemurs with the highest RP values (4) were those with the most unusual diets; *Archaeolemur* was highly frugivorous and likely able to exploit, like *Daubentonia*, structurally defended resources [6, 23], while *Hadropithecus* likely specialized on the leaves of succulent CAM plants [42], which could sustain it and provide needed water and nutrients without a dramatic reduction in activity levels throughout the prolonged dry season. The Archaeolemuridae had the largest brains of the subfossil lemurs, and semicircular canal data show that they were also the most active. While we cannot reconstruct age at first reproduction for these animals, we can draw inferences regarding the giant extinct lemurs on the basis of the data we have collected. Thus, we can infer that those extinct lemur species that were able to exploit resources that could provide reliable energy throughout the dry season also had relatively large brains, higher activity levels, and higher RP values. We can also assert that, among lemurs, brain size does better than body size in predicting RP. More research on how diet correlates with relative brain size and RP is clearly warranted.

The point is that lemurs display a variety of life history strategies each of which, in its own way, enables them to survive in an unpredictable climate with limited resource availability during particular times of the year. Godfrey et al. [43] discuss life history differences between lemurids and indriids, and these are relevant here as we can now show that they are tied to variation in RP. Indriids have relatively low RP values. They place a selective premium on adult survival (at the expense of rapid population recovery during a resource crunch) by being able to survive on low-quality resources. They are bet hedgers *par excellence*. Lemurids place a selective premium on being able to reproduce rapidly when conditions are right. They have higher adult mortality during resource crunches (given their greater need for high quality foods), but they begin reproducing earlier, are more likely to have twins, and have a higher maximum rate of population increase; thus population recovery is faster, when it is possible. RP values for lemurids tend to be higher than in indriids, but in comparison to other primates, they are still low.

With the single exception of *Daubentonia*, no analysis we performed makes the lemurs quantitatively or qualitatively resemble the anthropoids. Therefore, our end results confirm our first prediction that HHO characteristics seen previously in subfossil lemurs extend to lemurs in general. Also with regard to this prediction, we raised the possibility that strepsirrhines as a whole may share the RP patterns expressed in lemurs. While our statistics do show some differences between the whole strepsirrhine and lemur samples, it is difficult to extrapolate the non-lemur pattern in the overall picture since the species in this sample fall near the intersection of the anthropoid and strepsirrhine lines. Nevertheless, the differences we do see in our strepsirrhine vs. lemur analyses, the unusual RP pattern of *Daubentonia* (which may retain a primitive similarity to anthropoids, since anthropoid patterns resemble general mammal patterns as displayed in Bromage et al. [2]; see also below), and the maintenance of low values for RP despite a large range of body mass and life history variation within lemurs, all suggest that the pattern is likely lemuroid-specific. Data from more large-bodied lorisoids would be needed to confirm this. While our first prediction is supported, our results do not confirm our second prediction, that regressions reflecting metabolic rate will show differences among taxa that are less pronounced than differences seen when regressing body and brain mass. In other words, regressions for ln SCR for lemurs and strepsirrhines are no more similar to anthropoid regressions than they are for any other predictor variable.

When we examine some other general strepsirrhine characters associated with life history, we can begin to form explanations for this unusual expression of HHO physiology. We interpret the small RP range as being the likely result of energetic constraints imposed on lemuroids in particular by their highly unpredictable (and often highly seasonal) environments in Madagascar [44], which affects both males and females, there being no apparent sex difference in metabolic rates [45]. Lemurs are generally less active, maintain smaller home ranges, and have smaller brain mass than anthropoids of similar sizes [6]. Therefore, in keeping their brains small compared to anthropoids, lemurs are apportioning more of their limited energy budget to reproduction or to limiting adult mortality (as described above) rather than to maintaining expensive brain tissue. This is necessary because of the high potential for periodic drops in resource availability. In other words, lemurs can minimize calorie expenditure to varying degrees (depending on diet) to reduce adult mortality, which has the benefit that adults are able to successfully reproduce during “good” years (unlike “bad” years when infant mortality is high). The lemur HHO periodicity, being linked to brain mass (and probably diet) in these animals, then reflects that tradeoff with its restricted range of variation. If ln ECV is the best predictor of lemur HHO periodicity, the evidence suggests that a risk-aversion strategy restricting brain mass may be critical in explaining the RP patterns that lemuroids exhibit. Seen in the light of an ecology that restricts encephalization and metabolic rate across body sizes, the restricted range of variation in lemur RP is entirely expected.

Among anthropoids, RP is highly and significantly associated with encephalization and metabolism, and what is more, the slopes of these relationships are well above those of all other tissue and organ systems [12]: This means that as RP increases, brain size and BMR increase faster than all other anatomical systems. It is among these relationships that the metabolic ecology of strepsirrhines distinguishes itself.

The lemur species that colonized Madagascar is estimated to have weighed around 2000 g [46] although some researchers have posited weights that are considerably smaller [12,47]. Extant strepsirrhines weighing 2000 g or less generally have low RP values (around 2), and most are hypometabolic; therefore, one can presume that hypometabolism and a relatively low RP were plesiomorphic for lemurs. What is unusual about the Lemuroidea, however, is not that species weighing 2000 g or less have low RP values, but rather that, despite the evolution of gigantism in multiple lemuroid families, RP values remain low. This is associated with unusually low encephalization and depressed BMR in all including the largest extant, and apparently the very large-bodied extinct, lemurs.

The circumstances surrounding the radiation of the Lemuroidea are beginning to be understood. This superfamily comprises seven of the eight families of Malagasy lemurs; its common ancestor lived only 31 million years ago, tens of millions of years after initial colonization and the divergence of the Lemuroidea and the Daubentonioidea at around 50 million years ago [48]. The origin of the Lemuroidea immediately postdates the precipitous global temperature decline and concomitant extinction events associated with the initiation of the Antarctica ice cap (at the Eocene-Oligocene boundary, ~34 Ma) [48,49,50]. All seven lemuroid families (including the three megafaunal families that disappeared after humans arrived) originated during the Oligocene or Miocene (the Megaladapidae at 27.3±4.2 Ma, the Archaeolemuridae at 24±3.9 Ma, and the Palaeopropithecidae at 20.8±3.6 Ma) [40]. The rapid diversification of the Lemuroidea was coincident with the development of the monsoon system that affects Southern Asia and Madagascar today [51,52,53], and with the arrival and initial diversification on Madagascar of the major reptilian, avian and mammalian predators that are known to have preyed on giant subfossil lemurs [54]. This includes a large, horned crocodile (*Voay*, which likely arrived on Madagascar after the mid-Miocene), raptors belonging to the family Accipitridae (which likely arrived during the late Eocene or later), and the mammalian carnivorans, the Eupleridae (which likely arrived during the late Oligocene or early Miocene) (see summary in [50]). One can attribute the remarkable niche and body size variation in the Lemuroidea, then, to: (1) the availability of “vacant niches” following a major extinction event associated with precipitous atmospheric cooling at the E/O boundary; (2) increasing rainfall unpredictability associated with an intensifying Asian monsoon system; and (3) a rapidly changing predator guild. The retention in these animals of a restricted HHO of 2–4 days makes sense in the context of an environment changing over time to create strong seasonality and unpredictable resource availability.

The exception to the rule among extant lemurs is *Daubentonia*, which seems to have avoided the HHO constriction by exploiting more predictable and higher-quality resources. *Daubentonia* seems to have evolved an entirely different strategy for coping with the unpredictable environments of Madagascar. It is unknown when exactly this change occurred after the *Daubentonia* lineage diverged from the lineage leading to other lemurs some 50 Ma [40,55]. *Daubentonia* is able to take advantage of structurally defended resources such as wood-boring insects, which are generally available year-round [56]. Not surprisingly, these animals are relatively encephalized and have RP values that have the highest positive residuals and are near anthropoid regression lines in repeated analyses (Figs 3 and 4). They also have very large home ranges [37] and a relatively high BMR (S2 Table). This reinforces the argument that HHO evolves in response to ecological demands to help match growth, size, metabolism, and life history to those demands.

Among the extinct lemurs, it is the Archaeolemuridae (*Archaeolemur* and *Hadropithecus*) that have relatively long Retzius periodicities (low for anthropoids of similar body mass but well above the strepsirrhine regression line) (Fig 3). These are by no means the largest-bodied extinct lemurs, but they are relatively large-brained and were likely more active than other giant lemurs, judging from their SCR values [38]. *Archaeolemur* has been reconstructed as a *Daubentonia*-like omnivore; *Hadropithecus* did not have a similar diet [42] but appears to have specialized on staples that were reliably available even during the dry season in harsh environments.

In sum, our results show that HHO evolves in a complex manner in response to ecology and evolutionary history. In particular, these forces seem to have driven an unusual pattern in the evolution of HHO within the lemuroids as compared to other primates. The central factor governing HHO evolution seems to be energetics, which is expected given that life history theory in large part revolves around the adaptive apportioning of energetic resources over time. However, the energetics picture is not as simple as we initially predicted, since ln SCR (and therefore overall metabolic rate) correlates significantly but not strongly with ln RP in lemurs. Rather, lemurs seem to be risk averse, adaptively apportioning energy derived from limited, unpredictable resources to differing needs such as maximizing adult survival, maximizing population recovery rate, or increasing brain metabolism and activity levels. Differences in HHO expression between lemurs and anthropoids indicate that differing energetic needs among taxa can drive a variety of life history adaptations, even within a single order of mammals. Our study also shows that phylogenetic history is important in the evolution of specific HHO physiologies. However, the exceptions of *Archaeolemur* and *Hadropithecus* emphasize that diet is important to energetic constraints. Having been somewhat loosened from the hold of the ecological parameters that define the evolutionary patterns of the lemuroid group as a whole by having likely evolved to exploit resources different from those of other lemurs (as *Daubentonia* does), these taxa seem to have been able to move into life history spaces that more closely resemble those of monkeys and apes [57], although they do still fall well short of the anthropoid expression of these parameters. The ability of the HHO to evolve under selection which this demonstrates is important, because an HHO biology that can adjust to ecological factors may provide an important fitness advantage during shifting ecological conditions or when radiating into new niches. With these points in mind, future studies of HHO variation should be able to provide detailed insights into the nuances of life history evolution among numerous other mammalian taxa.

## Supporting Information

S1 Fig Phylogeny used in PGLS analyses. Includes all extant primates in the study and subfossil lemurs. See the main paper for details & references. To incorporate subfossil lemurs, branch lengths were calibrated as a chronogram. Anthropoid/strepsirrhine divergence date calibrated following Kistler et al. [40].
(PDF)

S1 Table Specimens and sources. All specimens that were used to generate new data (i.e., not taken from the literature) are listed here. Sources are listed as follows: UMass = University of Massachusetts uncatalogued, Jonah Ratsimbazafy Collection, Manombo Reserve; DLC = Duke Lemur Center; GDBP = Great Divide Basin Project; UM-APC = University of Massachusetts Anthropological Primate and Natural History Collections; ASU = Arizona State University; Newcastle = Newcastle University School of Dental Sciences; M = Montpelier II University; UZI = Universität Zürich-Irchel.
(PDF)

S2 Table Raw data table for our study. Preference was given to modal values for Retzius periodicity (RP) data whenever possible, since RP is expressed in multiples of 1 day; whenever data were available for only two or three specimens, mean values were used. However, the majority of species samples consist only of a single individual. For species with high sexual dimorphism, values for both males and females were included separately where available. RP and osteocyte data lacking a citation are newly published values generated by our study. References given in main paper; wherever possible, RP, mass, and ECV data drawn from the compilation in Bromage et al. [2] and Isler et al. [25]. All SCR data from Spoor et al. [29].
† = Extinct species
* = Modal value reported from literature
** = Data are presented for more than one individual for these species, but too few individuals have been sampled to incorporate a reliable mode value. For these species, statistical analyses incorporate means of the reported values.
(PDF)

S3 Table Correlations & regression statistics, raw values (without phylogenetic correction). **A**. Regression statistics (least squares) for ln RP & ln OD relationships. Significant relationships highlighted in bold. **B**. Bivariate correlations matrices for all variables. Significant correlations for RP & OD highlighted in bold. * = no data available for subfossil lemurs, so no separate analysis excluding subfossil species conducted; ** = sample too small for analysis.
(PDF)
