# Supplementary material for: Lemur Biorhythms and Life History Evolution
Source: PLoS One. 2015 Aug 12;10(8):e0134210. doi: 10.1371/journal.pone.0134210 (PMC4534448; doi:10.1371/journal.pone.0134210)

Figure S1. Phylogeny used in PGLS analyses. Includes all extant primates in the study and subfossil lemurs. See the main paper for details & references. To incorporate subfossil lemurs, branch lengths are calibrated as a chronogram. Anthropoid/strepsirrhine divergence date calibrated following Kistler et al. [40]

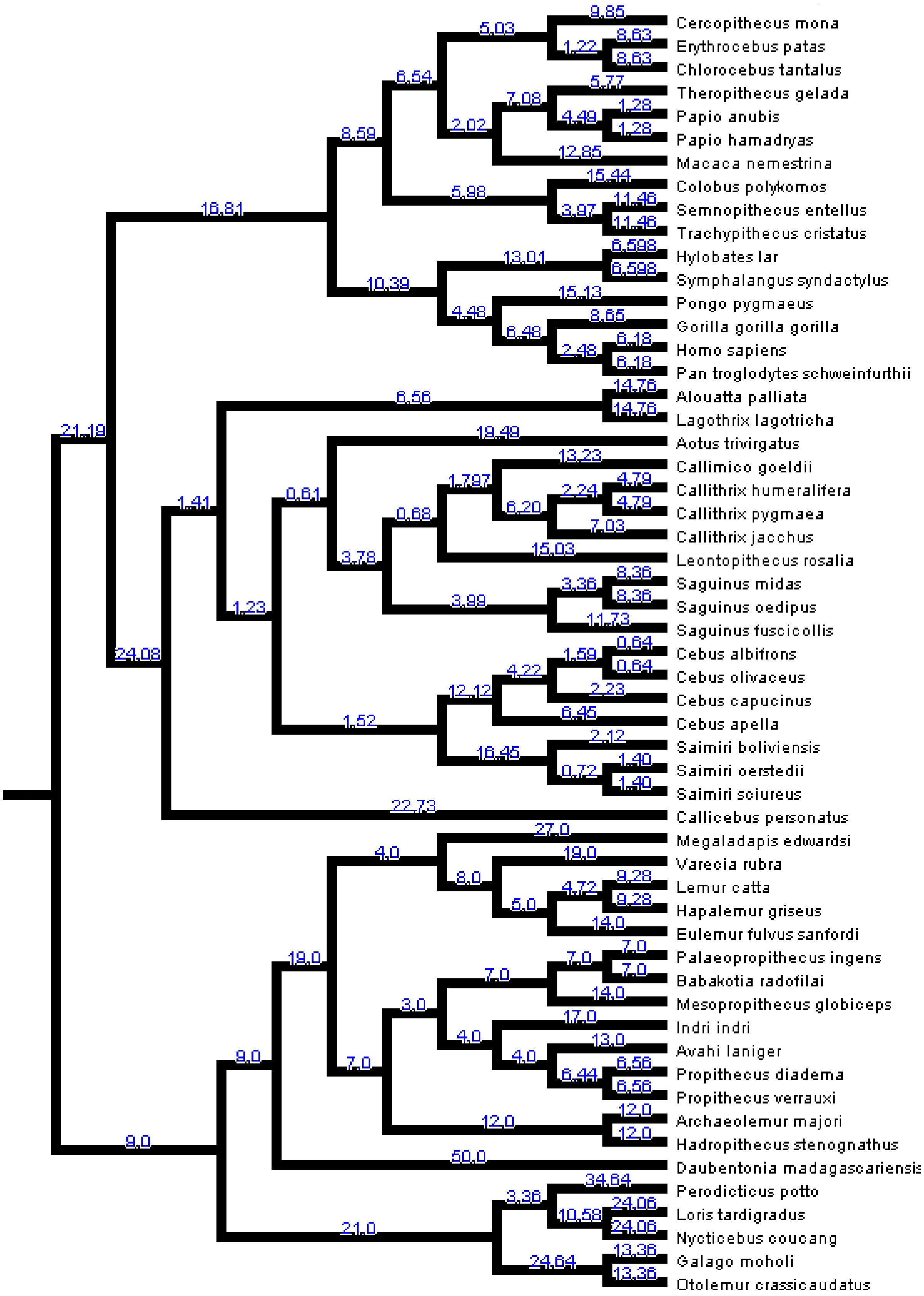

Supplement: S1 Fig — Includes all extant primates in the study and subfossil lemurs. See the main paper for details & references. To incorporate subfossil lemurs, branch lengths were calibrated as a chronogram. Anthropoid/strepsirrhine divergence date calibrated following Kistler et al. [40]. (PDF) [file pone.0134210.s001.pdf]
