# Supplementary material for: Lemur Biorhythms and Life History Evolution
Source: PLoS One. 2015 Aug 12;10(8):e0134210. doi: 10.1371/journal.pone.0134210 (PMC4534448; doi:10.1371/journal.pone.0134210)
Supplement: S1 Table — All specimens that were used to generate new data (i.e., not taken from the literature) are listed here. Sources are listed as follows: UMass = University of Massachusetts uncatalogued, Jonah Ratsimbazafy Collection, Manombo Reserve; DLC = Duke Lemur Center; GDBP = Great Divide Basin Project; UM-APC = University of Massachusetts Anthropological Primate and Natural History Collections; ASU = Arizona State University; Newcastle = Newcastle University School of Dental Sciences; M = Montpelier II University; UZI = Universität Zürich-Irchel. (PDF) [file pone.0134210.s002.pdf]

Table S1. Specimens and sources. All specimens which were used to generate new data (i.e., not taken from the literature) are listed here. Sources are listed as follows: UMass = University of Massachusetts uncatalogued, Jonah Ratsimbazafy Collection, Manombo Reserve; DLC = Duke Lemur Center; GDBP = Great Divide Basin Project; UM-APC = University of Massachusetts Anthropological Primate and Natural History Collections; ASU = Arizona State University; Newcastle = Newcastle University School of Dental Sciences; M = Montpelier II University; UZI = Universität Zürich-Irchel.

Taxon	Source	Specimen number	Specimen type
**strepsirrhines**			
Avahi laniger	UMass	N/A	femur
Babakotia radofilai	DLC	11832	tooth
Cantius abditus	GDBP	WMU-VP 359	tooth
Cantius abditus	GDBP	WMU-VP 654826	tooth
Cantius abditus	GDBP	WMU-VP 6541103	tooth
Cantius abditus	GDBP	WMU-VP 4587	tooth
Cantius abditus	GDBP	WMU-VP 371	tooth
Cantius abditus	GDBP	WMU-VP 363	tooth
Cantius abditus	GDBP	WMU-VP g501589	tooth
Daubentonia madagascariensis	DLC	"Poe"	tooth
Daubentonia madagascariensis	DLC	6514m	tooth, femur
Eulemur fulvus sanfordi	DLC	4564m	tooth, femur
Galago moholi	DLC & UM-APC	DLC 2018f<br>UM-APC 207	tooth, femur
Hapalemur griseus	DLC	1317f	tooth, femur
Indri indri	ASU	S26	tooth
Lemur catta	DLC & UM-APC	DLC 6530f<br>UM-APC 9	tooth
Lemur catta	DLC & UM-APC	DLC 6158f<br>UM-APC 253	tooth
Lemur catta	Newcastle	HT13-90	tooth
Leptadapis magnus	M	ACQ 6415	tooth
Loris tardigradus	DLC	2930m	tooth, femur
Nycticebus coucang	DLC & UM-APC	DLC 1949m<br>UM-APC 170	tooth, femur
Otolemur crassicaudatus	Newcastle	HT12-90	tooth

Otolemur crassicaudatus	Newcastle	HT 17-00	tooth
Perodicticus potto	UM-APC	163	tooth, femur
Propithecus coquereli	DLC	6845m	tooth, femur
Propithecus diadema	Newcastle	HT16-90	tooth
Varecia variegata rubra	DLC	5628f	tooth, femur
Varecia variegata	Newcastle	HT25-08	tooth
**anthropoids**			
Cercopithecus mona	Newcastle	HT01-10	tooth
Chlorocebus tantalus	Newcastle	HT06-02	tooth
Chlorocebus tantalus	Newcastle	HT07-02	tooth
Colobos polykomos	UZI	Co.p 3	tooth
Lagothrix poeppigii	University of Texas	N/A	tooth
Lagothrix poeppigii	University of Texas	N/A	tooth
Trachypithecus cristatus	Newcastle	HT19-05	tooth
