# Supplementary material for: Lemur Biorhythms and Life History Evolution
Source: PLoS One. 2015 Aug 12;10(8):e0134210. doi: 10.1371/journal.pone.0134210 (PMC4534448; doi:10.1371/journal.pone.0134210)
Supplement: S2 Table — Preference was given to modal values for Retzius periodicity (RP) data whenever possible, since RP is expressed in multiples of 1 day; whenever data were available for only two or three specimens, mean values were used. However, the majority of species samples consist only of a single individual. For species with high sexual dimorphism, values for both males and females were included separately where available. RP and osteocyte data lacking a citation are newly published values generated by our study. References given in main paper; wherever possible, RP, mass, and ECV data drawn from the compilation in Bromage et al. [2] and Isler et al. [25]. All SCR data from Spoor et al. [29]. † = Extinct species * = Modal value reported from literature ** = Data are presented for more than one individual for these species, but too few individuals have been sampled to incorporate a reliable mode value. For these species, statistical analyses incorporate means of the reported values. (PDF) [file pone.0134210.s003.pdf]

Table S2. Raw data table for our study. Preference was given to modal values for Retzius periodicity (RP) data whenever possible, since RP is expressed in multiples of 1 day; whenever data were available for only two or three specimens, mean values were used. However, the majority of species samples consist only of a single individual. For species with high sexual dimorphism, values for both males and females were included separately where available. RP and osteocyte data lacking a citation are newly published values generated by our study. References given in main paper; wherever possible, RP, mass, and ECV data drawn from the compilation in Bromage *et al.* [2] and Isler *et al.* [26]. All SCR data from Spoor *et al.* [30].

† =Extinct species
* = Modal value reported from literature
** = Data are presented for more than one individual for these species, but too few individuals have been sampled to incorporate a reliable mode value. For these species, statistical analyses incorporate means of the reported values.

genus & species	*RP, days (ln) [ref]*	*osteocyte density, per mm² (ln)*	*body mass, kg (ln) [ref]*	*endocranial volume, cc (ln) [ref]*	*semicircular canal radius, mm (ln)*	*basal metabolic rate, mLO₂/h (ln) [ref]*	*1st female reproduction, years (ln) [ref]*
strepsirrhines							
Archaeolemur majori†	4 (1.386) [24]	--	18.2 (2.901) [27]	93 (4.533) [27]	3 (1.090)	--	
Avahi laniger	2 (0.693)	632 (6.449)	1.32 0.278 [26]	9.86 (2.288) [26]	1.9 (0.642)	--	2.58 (0.948) [1]
Babakotia radofilai†	2 (0.693)	--	20.7 (3.03) [27]	48 (3.871) [27]	2.4 (0.875)	--	
Cantius abditus†	3** (1.099)	--	3 (1.099) [22]	--	--	--	
Daubentonia madagascariensis	3,4** (1.253)	549 (6.308)	2.59 (0.952) [26]	44.85 (3.803) [26]	2.4 (0.875)	1393.9 (7.240) [18]	3.5 (1.253) [38]

genus & species	*RP, days (ln) [ref]*	*osteocyte density, per mm^2 (ln)*	*body mass, kg (ln) [ref]*	*endocranial volume, cc (ln) [ref]*	*semicircular canal radius, mm (ln)*	*basal metabolic rate, mLO$_2$/h (ln) [ref]*	*1st female reproduction, years (ln) [ref]*
Eulemur sanfordi	3 (1.099)	724 (6.595)	2.292 (0.829) [26]	25.77 (3.249) [26]	2.2 (0.788)	940 (6.846) [18]	2.66 (0.978) [1]
Galago moholi	--	975 (6.882)	0.244 (-1.411) [2]	3.71 (1.311) [26]	1.5 (0.405)	198 (5.288) [18]	0.71 (-0.342) [1]
Hadropithecus stenognathus†	4 (1.386) [24]	--	35.4 (3.567) [27]	115 (4.745) [27]	2.7 (0.993)	--	--
Hapalemur griseus	2 (0.693)	857 (6.753)	0.709 (-0.344) [26]	14.09 (2.645) [26]	2 (0.693)	--	2.73 (1.004) [1]
Indri indri	2 (0.693)	--	6.84 (1.923) [26]	34.81 (3.560) [26]	2.7 (0.993)	--	8 (2.079) [36]
Lemur catta	2,3,3** (0.981)	--	2.21 (0.793) [26]	22.9 (3.131) [26]	2.1 (0.742)	651.4 (6.479) [18]	2.63 (0.967) [1]
Leptadapis magnus†	3 (1.099)	--	4 (1.386) [22]	21.7 (3.077) [29]	--	--	--
Loris tardigradus	3 (1.099)	476 (6.165)	0.193 (-1.645) [26]	5.87 (1.770) [26]	1.2 (0.182)	107.4 (4.677) [18]	1.5 (0.405) [36]
Megaladapis edwardsi†	3 (1.099) [13]	--	85.1 (4.444) [27]	137 (4.920) [27]	2.3 (0.833)	--	--

genus & species	*RP, days (ln) [ref]*	*osteocyte density, per mm²* *(ln)*	*body mass, kg (ln) [ref]*	*endocranial volume, cc (ln) [ref]*	*semicircular canal radius, mm (ln)*	*basal metabolic rate, mLO₂/h (ln) [ref]*	*1st female reproduction, years (ln) [ref]*
Mesopropithecus globiceps†	3 (1.099) [19]	--	11.3 (2.425) [27]	41 (3.714) [27]	2.3 (0.833)	--	--
Nycticebus coucang	2 (0.693)	562 (6.332)	0.679 (-0.387) [26]	10.41 (2.343) [26]	1.5 (0.405)	273 (5.609) [18]	--
Otolemur crassicaudatus	2,3** (0.916)	--	1.15 (0.140) [26]	11.78 (2.466) [26]	2 (0.693)	523 (6.260) [18]	2.21 (0.793) [36]
Palaeopropithecus ingens†	2 (0.693) [12]	--	41.5 (3.726) [27]	80 (4.382) [27]	1.9 (0.642)	--	--
Perodicticus potto	2,3** (0.916)	558 (6.324)	0.835 (-0.180) [26]	12.42 (2.519) [26]	1.7 (0.531)	326 (5.787) [18]	2.03 (0.708) [36]
Propithecus coquereli	2 (0.693)	619 (6.428)	4.052 (1.399) [26]	30.19 (3.408) [26]	--	1012.3 (6.920) [18]	--
Propithecus diadema	3 (1.099)	--	5.76 (1.751) [26]	40.92 (3.711) [26]	2.7 (0.993)	--	4.51 (1.506) [1]
Propithecus verreauxi	2 (0.693) [12]	--	2.95 (1.082) [26]	26.21 (3.266) [26]	2.3 (0.751)	670 (6.507) [18]	4.57 (1.519) [1]
Varecia variegata	3 (1.099)	471 (6.155)	3.52 (1.258) [26]	32.12 (3.469) [26]	2.2 (0.788)	603.6 (6.403) [18]	2.72 (1.001) [1]

genus & species	*RP, days (ln) [ref]*	*osteocyte density, per mm^2 (ln)*	*body mass, kg (ln) [ref]*	*endocranial volume, cc (ln) [ref]*	*semicircular canal radius, mm (ln)*	*basal metabolic rate, mLO$_2$/h (ln) [ref]*	*1st female reproduction, years (ln) [ref]*
anthropoids							
Afropithecus turkanensis†	8<br>(2.079)<br>[2]	--	34.5<br>(3.541)<br>[2]	--	--	--	
Alouatta sp.	6<br>(1.792)<br>[2]	--	6.415<br>(1.859)<br>[26]	54.3<br>(3.995)<br>[26]	2.4<br>(0.875)	2055<br>(7.628)<br>[32]	3.8<br>(1.335)<br>[7]
Aotus sp.	3<br>(1.099)<br>[2]	--	0.91<br>(-0.094)<br>[26]	17.74<br>(2.876)<br>[26]	2<br>(0.693)	496<br>(6.207)<br>[23]	2.42<br>(0.884)<br>[7]
Callicebus personatus	3<br>(1.099)<br>[2]	--	1.305<br>(0.266)<br>[26]	19.84<br>(2.988)<br>[26]	--	--	--
Callimico goeldii	3<br>(1.099)<br>[2]	--	0.389<br>(-0.944)<br>[26]	11.24<br>(2.419)<br>[26]	1.6<br>(0.470)	--	1.3<br>(0.262)<br>[7]
Callithrix humeralifer	3<br>(1.099)<br>[2]	--	0.35<br>(-1.040)<br>[2]	8.2<br>(2.104)<br>[26]	--	--	1.6<br>(0.470)<br>[7]
Callithrix jacchus	1<br>(0)<br>[2]	--	0.257<br>(-1.359)<br>[26]	7.21<br>(1.975)<br>[26]	1.5<br>(0.405)	154<br>(5.037)<br>[23]	1.67<br>(0.513)<br>[7]
Cebuella pygmaea	1<br>(0)<br>[2]	--	0.123<br>(-2.096)<br>[26]	4.24<br>(1.444)<br>[26]	--	89<br>(4.489)<br>[23]	1.9<br>(0.642)<br>[7]
Cebus albifrons (M)	6<br>(1.792)<br>[2]	--	2.48<br>(0.908)<br>[26]	64.11<br>(4.161)<br>[26]	--	--	4<br>(1.386)<br>[7]

genus & species	RP, days (ln) [ref]	osteocyte density, per mm² (ln)	body mass, kg (ln) [ref]	endocranial volume, cc (ln) [ref]	semicircular canal radius, mm (ln)	basal metabolic rate, mLO_2/h (ln) [ref]	1st female reproduction, years (ln) [ref]
Cebus albifrons (F)	5 (1.609) [2]	--	1.814 (0.596) [26]	63.24 (4.147) [26]	--	--	4 (1.386) [7]
Cebus apella (M)	5 (1.609) [2]	--	3.38 (1.218) [26]	68.94 (4.233) [26]	2.2 (0.788)	--	5.78 (1.754) [7]
Cebus apella (F)	4 (1.386) [2]	--	2.49 (0.912) [26]	64.3 (4.164) [26]	2.2 (0.788)	--	5.78 (1.754) [7]
Cebus capucinus (M)	6 (1.792) [2]	--	3.267 (1.184) [26]	73.13 (4.292) [26]	--	--	4 (1.386) [7]
Cebus capucinus (F)	4 (1.386) [2]	--	3.267 (1.184) [26]	72.12 (4.278) [26]	--	--	4 (1.386) [7]
Cebus olivaceus	4 (1.386) [2]	--	2.974 (1.090) [26]	69.84 (4.246) [26]	--	--	6 (1.792) [7]
Cercopithecus mona	4 (1.386)	--	3.313 (1.198) [26]	61.84 (4.125) [26]	2.3 (0.833)	--	4 (1.386) [34]
Chlorocebus tantalus	3,4** (1.253)	--	4.082 (1.407) [26]	56.77 (4.039) [26]	2.3 (0.833)	--	4 (1.386) [37]
Colobus polykomos	4 (1.386)	--	8.654 (2.158) [26]	73.38 (4.296) [26]	2.9 (1.065)	--	8.5 (2.140) [35]

genus & species	***RP, days (ln) [ref]***	***osteocyte density, per mm^2 (ln)***	***body mass, kg (ln) [ref]***	***endocranial volume, cc (ln) [ref]***	***semicircular canal radius, mm (ln)***	***basal metabolic rate, mLO$_2$/h (ln) [ref]***	***1st female reproduction, years (ln) [ref]***
Dryopithecus laietanus†	7 (1.946) [2]	--	23 (3.135) [2]	--	--	--	--
Erythrocebus patas	4 (1.386) [2]	--	4.8 (1.569) [26]	93.88 (4.542) [26]	2.5 (0.916)	1068 (6.974) [32]	3 (1.099) [35]
Gigantopithecus blacki†	11 (2.398) [2]	--	300 (5.704) [2]	--	--	--	--
Gorilla gorilla (M)	10* (2.303) [2]	--	169.3 (5.132) [26]	524.92 (6.263) [26]	3 (1.099)	--	10.3 (2.332) [1]
Gorilla gorilla (F)	9* (2.197) [2]	--	75.7 (4.327) [26]	455.89 (6.122) [26]	3 (1.099)	--	10.3 (2.332) [1]
Graecopithecus freybergi†	8 (2.079) [2]	--	63 (4.143) [2]	--	--	--	--
Homo sapiens (M)	8* (2.079) [2]	--	60.2 (4.098) [26]	1409 (7.251) [26]	2.9 (1.065)	12500 (9.433) [23]	19.3 (2.961) [35]
Homo sapiens (F)	8* (2.079) [2]	--	53.6 (3.982) [26]	1409 (7.251) [26]	2.9 (1.065)	12500 (9.433) [23]	19.3 (2.961) [35]
Hylobates lar	4 (1.386) [2]	--	5.6 (1.723) [26]	102.11 (4.626) [26]	2.6 (0.956)	--	9.8 (2.282) [33]

genus & species	*RP, days (ln) [ref]*	*osteocyte density, per mm^2 (ln)*	*body mass, kg (ln) [ref]*	*endocranial volume, cc (ln) [ref]*	*semicircular canal radius, mm (ln)*	*basal metabolic rate, mLO_2/h (ln) [ref]*	*1st female reproduction, years (ln) [ref]*
Hylobates syndactylus	4,5** (1.504) [2]	--	10.7 (2.370) [26]	123.83 (4.819) [26]	2.8 (1.030)	--	9 (2.197) [33]
Lagothrix poeppigii	5,6** (1.705)	--	9.072 (2.205) [26]	99.23 (4.597) [26]	2.5 (0.916)	--	5 (1.609) [36]
Leontopithecus rosalia	3 (1.099) [2]	--	0.628 (-0.465) [26]	12.65 (2.538) [26]	1.5 (0.405)	382 (5.945) [23]	2.4 (0.875) [7]
Macaca nemestrina	4 (1.386) [2]	--	7.8 (2.054) [26]	106.04 (4.664) [26]	2.5 (0.916)	--	3.9 (1.361) [1]
Pan troglodytes	6 (1.792) [2]	--	35.8 (3.578) [26]	377.57 (5.934) [26]	2.7 (0.993)	9070 (9.113) [23]	13.9 (2.632) [1]
Papio hamadryas hamadryas	7 (1.946) [2]	--	9.4 (2.241) [26]	149.93 (5.010) [26]	2.7 (0.993)	--	6.1 (1.808) [36]
Papio hamadryas anubis	7 (1.946) [2]	--	21 (3.044) [26]	168.64 (5.128) [26]	2.7 (0.993)	2778 (7.929) [32]	4.5 (1.504) [1]
Pongo pygmaeus (M)	10* (2.303) [2]	--	78.3 (4.361) [26]	427.51 (6.058) [26]	2.6 (0.956)	4860 (8.489) [21]	14.32 (2.662) [1]
Pongo pygmaeus (F)	10* (2.303) [2]	--	35.8 (3.578) [26]	338.51 (5.825) [26]	2.6 (0.956)	4860 (8.489) [21]	14.32 (2.662) [1]

genus & species	*RP, days (ln) [ref]*	*osteocyte density, per mm^2 (ln)*	*body mass, kg (ln) [ref]*	*endocranial volume, cc (ln) [ref]*	*semicircular canal radius, mm (ln)*	*basal metabolic rate, mLO$_2$/h (ln) [ref]*	*1st female reproduction, years (ln) [ref]*
Proconsul heseloni†	5 (1.609) [2]	--	10.5 (2.351) [2]	167 (5.118) [31]	--	--	--
Proconsul nyanzae†	6 (1.792) [2]	--	35 (3.555) [2]	--	--	--	--
Saguinus fuscicollis	2 (0.693) [2]	--	0.373 (-0.986) [26]	7.92 (2.069) [26]	--	--	2.33 (0.846) [7]
Saguinus nigricollis	2 (0.693) [2]	--	0.45 (-0.734) [25]	8.9 (2.186) [25]	--	--	2.33 (0.846) [7]
Saguinus oedipus	1 (0) [2]	--	0.43 (-0.844) [26]	9.71 (2.273) [26]	1.5 (0.405)	--	1.89 (0.637) [7]
Saimiri boliviensis	3 (1.099) [2]	--	0.7 (-0.357) [2]	25.64 (3.244) [26]	--	--	--
Saimiri oerstedii	3 (1.099) [2]	--	0.829 (-0.188) [26]	24.97 (3.218) [26]	--	--	--
Saimiri sciureus	3 (1.099) [2]	--	0.852 (-0.16) [26]	23.62 (3.162) [26]	1.9 (0.642)	592 (6.383) [23]	2.5 (0.916) [7]
Semnopithecus entellus priam	5 (1.609) [2]	--	9.9 (2.293) [26]	110.93 (4.709) [26]	2.5 (0.916)	--	4.5 (1.504) [33]

genus & species	***RP, days (ln) [ref]***	***osteocyte density, per mm² (ln)***	***body mass, kg (ln) [ref]***	***endocranial volume, cc (ln) [ref]***	***semicircular canal radius, mm (ln)***	***basal metabolic rate, mLO₂/h (ln) [ref]***	***1st female reproduction, years (ln) [ref]***
Theropithecus gelada (M)	7 (1.946) [2]	--	20.5 (3.020) [26]	142.13 (4.957) [26]	2.5 (0.916)	--	4 (1.386) [36]
Theropithecus gelada (F)	7 (1.946) [2]	--	13.6 (2.610) [26]	124.52 (4.824) [26]	2.5 (0.916)	--	4 (1.386) [36]
Theropithecus oswaldi†	6 (1.792) [28]	--	25 (3.219) [20]	--	--	--	--
Trachypithecus cristatus	4 (1.386)	--	6.3 (1.841) [26]	57.86 (4.058) [26]	--	--	4 (1.386) [1]
