# Supplementary material for: Lemur Biorhythms and Life History Evolution
Source: PLoS One. 2015 Aug 12;10(8):e0134210. doi: 10.1371/journal.pone.0134210 (PMC4534448; doi:10.1371/journal.pone.0134210)
Supplement: S3 Table — A. Regression statistics (least squares) for ln RP & ln OD relationships. Significant relationships highlighted in bold. B. Bivariate correlations matrices for all variables. Significant correlations for RP & OD highlighted in bold. * = no data available for subfossil lemurs, so no separate analysis excluding subfossil species conducted; ** = sample too small for analysis. (PDF) [file pone.0134210.s004.pdf]

Table S3. Correlations & regression statistics, raw values (without phylogenetic correction). A. Regression statistics (least squares) for ln RP & ln OD relationships. Significant relationships highlighted in bold. **B.** Bivariate correlations matrices for all variables. Significant correlations for RP & OD highlighted in bold. * = no data available for subfossil lemurs, so no separate analysis excluding subfossil species conducted; ** = sample too small for analysis.

A.	***sample***	***r^2***	***p***	***n***	***B***	***standard error***	***Beta***	***95% confidence interval***	
								lower bound	***upper bound***
ln Body Mass vs ln RP	***primates***	***0.552***	***<0.01***	***70***	***0.234***	***0.260***	***0.743***	***0.183***	***0.284***
	anthropoids	***0.806***	***<0.01***	***48***	***0.275***	***0.020***	***0.898***	***0.235***	***0.315***
	strepsirrhines	0.053	0.30	22	0.036	0.034	0.230	-0.035	0.107
	lemurs	0.298	0.26	16	0.058	0.50	0.298	-0.049	0.165
	streps. no subfossil	0.007	0.75	16	0.018	0.057	0.085	-0.105	0.141
	non-lemur streps.	0.059	0.64	6	0.036	0.072	0.244	-0.163	0.236
ln ECV vs ln RP	***primates***	***0.706***	***<0.01***	***64***	***0.347***	***0.028***	***0.840***	***0.290***	***0.404***
	anthropoids	***0.776***	***<0.01***	***43***	***0.360***	***0.030***	***0.881***	***0.299***	***0.420***
	strepsirrhines	0.162	0.07	21	0.114	0.059	0.403	-0.010	0.239
	lemurs	***0.301***	***0.03***	***16***	***0.202***	***0.082***	***0.549***	***0.026***	***0.378***
	streps. no subfossil	0.066	0.36	16	0.087	0.091	0.256	-0.109	0.283
	non-lemur streps.	0.003	0.93	6	0.019	0.207	0.052	-0.640	0.677
ln SCR vs ln RP	***primates***	***0.456***	***<0.01***	***47***	***1.721***	***0.281***	***0.675***	***1.156***	***2.286***
	anthropoids	***0.686***	***<0.01***	***28***	***2.280***	***0.303***	***0.828***	***1.658***	***2.902***
	strepsirrhines	0.120	0.15	19	0.383	0.252	0.346	-0.149	0.914
	lemurs	***0.328***	***0.03***	***15***	***1.118***	***0.443***	***0.573***	***0.161***	***2.074***
	streps. no subfossil	0.005	0.82	14	0.061	0.269	0.068	-0.531	0.653
	non-lemur streps.	0.131	0.639	5	-0.278	0.508	-0.361	-2.464	1.907

ln AFR vs ln RP	***primates***	***0.507***	***<0.01***	***51***	***0.471***	***0.085***	***0.712***	***0.431***	***0.741***
	anthropoids	***0.563***	***<0.01***	***39***	***0.631***	***0.091***	***0.750***	***0.446***	***0.817***
	strepsirrhines*	0.099	0.319	12	-0.144	0.37	-0.315	-0.450	0.162
	lemurs	0.072	0.486	9	-0.158	0.215	-0.268	-0.666	0.350
	non-lemur streps.**	-	-	-	-	-	-	-	-
ln Body mass vs ln OD	***primates***	-	-	-	-	-	-	-	-
	anthropoids	-	-	-	-	-	-	-	-
	strepsirrhines	0.068	0.47	10	-0.059	0.077	-0.260	-0.236	0.119
	lemurs	0.566	0.08	6	-0.240	0.105	-0.752	-0.531	0.052
ln ECV vs ln OD	***primates***	-	-	-	-	-	-	-	-
	anthropoids	-	-	-	-	-	-	-	-
	strepsirrhines	0.128	0.31	10	-0.107	0.099	-0.358	-0.335	0.121
	lemurs	0.315	0.25	6	-0.207	0.153	-0.561	-0.631	0.217
ln SCR vs ln OD	***primates***	-	-	-	-	-	-	-	-
	anthropoids	-	-	-	-	-	-	-	-
	strepsirrhines	0.003	0.89	9	0.059	0.422	0.053	-0.938	1.057
	lemurs	0.233	0.41	5	-1.233	1.291	-0.483	-5.342	2.877
ln AFR vs ln OD	***primates***	-	-	-	-	-	-	-	-
	anthropoids	-	-	-	-	-	-	-	-
	strepsirrhines	0.185	0.288	8	-0.228	0.195	-0.430	-0.706	0.250
	lemurs	0.108	0.590	5	-0.623	1.035	0.328	-3.918	2.671

B.		***ln RP***	***ln body mass***	***ln ECV***	***ln SCR***	***ln OD***	***ln AFR***
primates							
ln RP	r	1	***0.743***	***0.840***	***0.675***	-	***0.712***
	r^2	1	***0.552***	***0.706***	***0.456***	-	***0.508***
	p	-	***< 0.01***	***< 0.01***	***< 0.01***	-	***< 0.01***
	n	74	***70***	***64***	***47***	-	***51***
ln body mass	r	***0.743***	1	0.916	0.837	-	0.881
	r^2	***0.552***	1	0.839	0.701	-	0.776
	p	***< 0.01***	-	< 0.01	< 0.01	-	< 0.01
	n	***70***	74	67	50	-	54
ln ECV	r	***0.840***	0.916	1	0.836	-	0.909
	r^2	***0.706***	0.839	1	0.699	-	0.826
	p	***< 0.01***	< 0.01	-	< 0.01	-	< 0.01
	n	***64***	67	67	49	-	53
ln SCR	r	***0.675***	0.837	0.836	1	-	0.831
	r^2	***0.456***	0.701	0.699	1	-	0.691
	p	***< 0.01***	< 0.01	< 0.01	-	-	< 0.01
	n	***47***	50	49	57	-	50
ln AFR	r	***0.712***	0.881	0.909	0.800	-	1
	r^2	***0.508***	0.776	0.826	0.640	-	1
	p	***< 0.01***	< 0.01	< 0.01	< 0.01	-	-
	n	***51***	54	53	50	-	61
anthropoids							
ln RP	r	1	***0.898***	***0.881***	***0.828***	-	***0.764***
	r^2	1	***0.806***	***0.776***	***0.686***	-	***0.584***
	p	-	***< 0.01***	***< 0.01***	***< 0.01***	-	***< 0.01***
	n	52	***48***	***43***	***28***	-	***38***
ln body mass	r	***0.898***	1	0.961	0.904	-	0.882
	r^2	***0.806***	1	0.924	0.817	-	0.778
	p	***< 0.01***	-	< 0.01	< 0.01	-	< 0.01
	n	***48***	51	45	30	-	40

ln ECV	r	***0.881***	0.961	1	0.886	-	0.922
	r^2	***0.776***	0.924	1	0.785	-	0.850
	p	***< 0.01***	< 0.01	-	< 0.01	-	< 0.01
	n	***43***	45	45	29	-	39
ln SCR	r	***0.828***	0.904	0.886	1	-	0.796
	r^2	***0.686***	0.817	0.785	1	-	0.634
	p	***< 0.01***	< 0.01	< 0.01	-	-	< 0.01
	n	***28***	30	29	31	-	30
ln AFR	r	***0.747***	0.869	0.911	0.804	-	1
	r^2	***0.558***	0.755	0.829	0.646	-	1
	p	***< 0.01***	< 0.01	< 0.01	< 0.01	-	-
	n	***38***	39	38	30	-	40
strepsirrhines							
ln RP	r	1	0.230	0.403	0.346	-0.475	-0.315
	r^2	1	0.053	0.162	0.120	0.226	0.099
	p	-	0.303	0.070	0.147	0.197	0.319
	n	22	22	21	19	9	12
ln body mass	r	0.230	1	0.959	0.732	-0.260	0.875
	r^2	0.053	1	0.920	0.536	0.068	0.766
	p	0.303	-	< 0.01	< 0.01	0.468	< 0.01
	n	22	23	22	20	10	13
ln ECV	r	0.403	0.959	1	0.791	-0.358	0.850
	r^2	0.162	0.920	1	0.626	0.128	0.723
	p	0.070	< 0.01	-	< 0.01	0.310	< 0.01
	n	21	22	22	20	10	13
ln SCR	r	0.346	0.732	0.791	1	-0.053	0.836
	r^2	0.120	0.536	0.626	1	0.003	0.699
	p	0.147	< 0.01	< 0.01	-	0.892	< 0.01
	n	19	20	20	26	9	19
ln OD	r	-0.475	-0.260	-0.358	0.053	1	-0.430
	r^2	0.226	0.068	0.128	0.003	1	0.185
	p	0.197	0.468	0.310	0.892	-	0.288
	n	9	10	10	9	10	19

ln AFR	r	-0.315	0.875	0.850	0.836	-0.430	1
	r^2	0.099	0.766	0.723	0.699	0.185	1
	p	0.319	< 0.01	< 0.01	< 0.01	0.288	-
	n	12	13	13	19	19	19
lemurs							
ln RP	r	1	0.298	***0.549***	***0.573***	-0.521	-0.268
	r^2	1	0.089	***0.301***	***0.328***	0.271	0.072
	p	-	0.263	***0.028***	***0.025***	0.289	0.486
	n	16	16	***16***	***15***	6	9
ln body mass	r	0.298	1	0.921	0.369	-0.752	0.730
	r^2	0.089	1	0.848	0.136	0.566	0.533
	p	0.263	-	< 0.01	0.176	.084	.025
	n	16	16	16	15	6	9
ln ECV	r	***0.549***	0.921	1	0.536	-0.561	0.517
	r^2	***0.301***	0.848	1	0.287	0.315	0.267
	p	***0.028***	< 0.01	-	0.040	0.247	0.154
	n	***16***	16	16	15	6	9
ln SCR	r	***0.573***	0.369	0.536	1	-0.483	0.837
	r^2	***0.328***	0.136	0.287	1	0.233	0.701
	p	***0.025***	0.176	0.040	-	0.410	<0.01
	n	***15***	15	15	19	5	13
ln OD	r	-0.521	-0.752	-0.561	-0.483	1	-.328
	r^2	0.271	0.566	0.315	0.233	1	0.108
	p	0.289	.084	0.247	0.410	-	0.590
	n	6	6	6	5	6	5
ln AFR	r	-0.268	0.730	0.517	0.837	-.328	1
	r^2	0.072	0.533	0.267	0.701	0.108	1
	p	0.486	.025	0.154	<0.01	0.590	-
	n	9	9	9	13	5	13
